# A novel lithium copper iron phosphate with idealized formula Li_5_Cu_2_^2+^Fe^3+^(PO_4_)_4_: crystal structure and distribution of defects

**DOI:** 10.1107/S1600536811011755

**Published:** 2011-04-07

**Authors:** Shailesh Upreti, Olga V. Yakubovich, Natasha A. Chernova, M. Stanley Whittingham

**Affiliations:** a Chemistry and Materials, SUNY Binghamton, Binghamton, NY, USA; b Department of Geology, Moscow State University, Moscow, Russian Federation

## Abstract

Gray–green single crystals were obtained under high-pressure, high-temperature hydro­thermal conditions. A refinement of atom occupancies gave the composition Li_3.68_Cu^2+^Fe^3+^(Cu_0.55_Li_0.45_)_2_Fe^2+^_0.15_(PO_4_)_4_. The structure is built from triplets of edge-sharing (Cu,Li)O_5_–FeO_6_–(Cu,Li)O_5_ polyhedra, CuO_4_ quadrilaterals and PO_4_ tetra­hedra. In the (Cu,Li)O_5_ polyhedra the Cu and Li positions are statistically occupied in a 0.551 (2):0.449 (2) ratio. Both FeO_6_ and CuO_4_ polyhedra exhibit 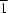 symmetry. The positions of additional Li atoms with vacancy defects are in the inter­stices of the framework.

## Related literature

For a related structure, see: Yakubovich *et al.* (2006 ▶). For related materials with low concentration of Cu atoms at Fe sites, see: Amine *et al.* (2000 ▶); Heo *et al.* (2009 ▶); Ni *et al.* (2005 ▶); Yang *et al.* (2009 ▶). For information on bond-valence calculations, see: Pyatenko (1972 ▶).

## Experimental

### 

#### Crystal data

Cu_2.10_Fe_1.15_Li_4.59_(PO_4_)_4_
*M* *_r_* = 609.63
Triclinic, 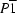

*a* = 4.8950 (14) Å
*b* = 7.847 (2) Å
*c* = 8.388 (2) Å
α = 69.472 (5)°
β = 89.764 (6)°
γ = 75.501 (5)°
*V* = 290.88 (13) Å^3^
*Z* = 1
Mo *K*α radiation
μ = 5.87 mm^−1^
*T* = 298 K
0.27 × 0.23 × 0.19 mm

#### Data collection

Bruker SMART APEX diffractometer
Absorption correction: multi-scan (*SADABS*; Bruker, 2000 ▶) *T*_min_ = 0.235, *T*_max_ = 0.332
100441 measured reflections
1775 independent reflections
1647 reflections with *I* > 2σ(*I*)
*R*_int_ = 0.023

#### Refinement

*R*[*F* ^2^ > 2σ(*F* ^2^)] = 0.031
*wR*(*F* ^2^) = 0.086
*S* = 1.20
1708 reflections
143 parameters
Δρ_max_ = 0.66 e Å^−3^
Δρ_min_ = −0.63 e Å^−3^

### 

Data collection: *SMART* (Bruker, 2001 ▶); cell refinement: *SAINT* (Bruker, 2002 ▶); data reduction: *SAINT*; program(s) used to solve structure: *SHELXS97* (Sheldrick, 2008 ▶); program(s) used to refine structure: *SHELXL97* (Sheldrick, 2008 ▶); molecular graphics: *DIAMOND* (Brandenburg, 2006 ▶); software used to prepare material for publication: *publCIF* (Westrip, 2010 ▶).

## Supplementary Material

Crystal structure: contains datablocks I, global. DOI: 10.1107/S1600536811011755/pk2312sup1.cif

Structure factors: contains datablocks I. DOI: 10.1107/S1600536811011755/pk2312Isup2.hkl

Additional supplementary materials:  crystallographic information; 3D view; checkCIF report

## Figures and Tables

**Table 1:** Selected bond lengths (Å)

Cu1—O4^i^	1.936 (2)
Cu1—O6	1.9430 (19)
Fe1—O1	1.931 (2)
Fe1—O5	2.038 (2)
Fe1—O2	2.041 (2)
Cu2—O8	1.969 (2)
Cu2—O7	1.998 (2)
Cu2—O6	2.003 (2)
Cu2—O5	2.075 (2)
Cu2—O2^ii^	2.171 (2)
Li1—O3	1.967 (8)
Li1—O4^iii^	2.028 (7)
Li1—O8^iv^	2.045 (7)
Li1—O3^v^	2.124 (8)
Fe2—O3	1.891 (6)
Fe2—O8^vi^	2.063 (6)
Fe2—O7^vii^	2.133 (7)
Fe2—O6^vi^	2.236 (7)
Fe2—O4^iii^	2.334 (6)
Li3—O7	1.909 (8)
Li3—O3^viii^	1.916 (7)
Li3—O2^viii^	2.183 (11)
Li3—O8^ix^	2.183 (10)

Symmetry codes: (i) 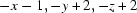; (ii) 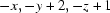; (iii) 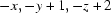; (iv) 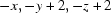; (v) 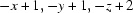; (vi) 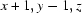; (vii) 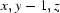; (viii) 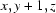; (ix) 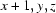.

**Fig. 1.**
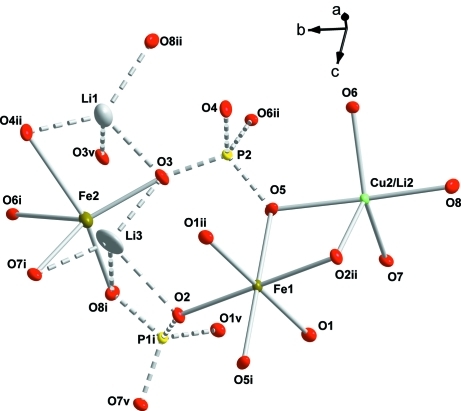
The main structural elements of the title compound. Displacement ellipsoids are drawn at the 50% probability level. [Symmetry codes: (i) x, y - 1, z; (ii) -x, 1 - y, 1 - z; (v) 1 - x, 1 - y, 1 - z].

**Fig. 2.**
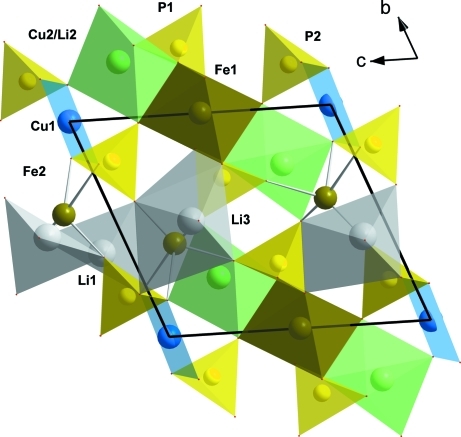
The crystal structure of the title compound projected onto the plane cb.

**Fig. 3.**
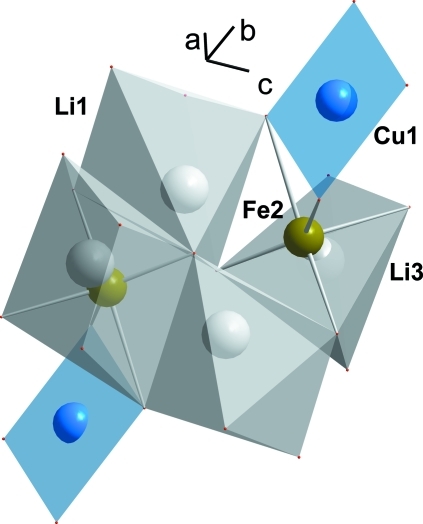
The structure fragment showing the groups of five-vertex Li polyhedra sharing edges.

## supplementary crystallographic information

### Comment

There has been much interest in understanding the chemical and physical behavior of a new class of materials that shows reversible intercalation of lithium in the crystalline lattice for use in the next generation of Li ion batteries.  Here we report a new type of Li containing solid which could be of great interest to electrochemists.

The asymmetric unit of the triclinic structure (Fig. 1) includes two tetrahedral P sites, both on the general position. The Cu1 – O distances around the square–planar Cu^2+^ cation at the center of symmetry (1*d* site) are 1.936 (2) and 1.943 (2) Å. Fe^3+^ cations in 1*b* Wyckoff site are surrounded by six O atoms, forming octahedral configuration with Fe—O bond lengths in the interval 1.931 (2) – 2.041 (2) Å.  The cation-anion distances in five-vertex polyhedra, occupied by Cu and Li atoms in nearly equivalent amounts change from 1.969 (2) to 2.171 (2) Å; thus, the mixed occupation of the polyhedron by Cu^2+^ and Li^+^ cations explains why the Jahn-Teller distorton of the polyhedron is not so evident.  Two Li sites with vacancy defects adopt five-vertex coordination, each with four closest oxygen atoms (Table 1), and one oxygen atom at longer distances of 2.739 (11) Å (Li3 –O8) and 2.778 (8) Å (Li1 – O3). In addition, a position of low occupancy for Fe^2+^ (Fe2) atoms has been found at 0.99 (1) Å from the Li3 site. Bond-valance sum data (Pyatenko, 1972) are consistent with the assumed oxidation state of Cu and Fe.

The basic features of the crystal structure consist of triplets of edge sharing (Cu,Li)2 – Fe1 –(Cu,Li)2 polyhedra (Fig.2) and Cu1 quadrilaterals, that form a three-dimensional framework by sharing oxygen vertices. The PO_4_ tetrahedra strengthen this framework by sharing all vertices with Fe1 octahedra and/or (Cu,Li)2 polyhedra (P1), while P2 tetrahedron shares one vertex with Fe1 and (Cu,Li)2 polyhedra, two vertices with Cu1 quadrilaterals, and one vertex (O3) remains unshared with the cationic framework and participates in the coordination of Li atoms (Table 2). Li1 and Li3 atoms occupy interstices of the structure; they form tetra groups of five-vertex polyhedra sharing edges (Fig.3).  The structure may be described using an idealized formula Li_5_Cu^2+^_2_Fe^3+^(PO_4_)_4_; a similar lithium saturated iron phosphate with isomorphous and vacancy defects in the position of Li atoms, having an idealized formula Li_5_Fe^3+^(PO_4_)_2_F_2_ was studied in (Yakubovich *et al.*, 2006). There are many reports in literature for crystal structures with low concentration of Cu atoms in Fe sites (Amine *et al.*, 2000; Heo *et al.*, 2009, Yang *et al.*, 2009, Ni *et al.*, 2005), however, the present structure seems to be a rare example of Cu rich three-dimensional matrix with Li^+^ ions in the interstices.

### Experimental

Single crystals were grown under high-temperature high-pressure hydrothermal conditions in the LiH_2_PO_4_—Fe_2_O_3_—H_3_PO_4_ system.  Fe_2_O_3_ and LiH_2_PO_4_, weight ratio 5:1, were placed in a copper ampule of 120 ml volume with 5, 10, 20, 30 or 40% water solution of H_3_PO_4_.  The reaction was conducted at 400 °C, 1000 bar (1 bar = 10 ^5^ Pa) for 100 h.  The reaction product was a mixture of brown, grayish-green, and blue-green crystals, white powder and copper chunks in a ratio dependant upon phosphoric acid concentration.  The crystals were hand picked under an optical microscope, washed with water and isopropyl alcohol, dried and subjected to single-crystal *x*-ray diffraction analysis.  The obtained crystals were also analyzed with a Jeol 8900 Electron Microprobe for EDS elemental content.  The conditions were optimized with an acceleration potential of 15 kV and acurrent of 10 mA. The average result of 15 analyses showed the Cu: Fe: P ratio equal to 0.47: 0.28: 1, which is close to the ratio 0.52: 0.29: 1 determined from single-crystal X-ray refinement.

### Refinement

Refinement of site occupancies showed that Cu2 and Li2 atoms share one position in the structure, in the proportion of 0.551 (2): 0.449. During the refinement, the displacement parameters of Cu2 and Li2 were constrained to be equal. The oxidation states of Fe atoms in 1*b* and 2*i* Wyckoff sites were fixed in accordance with Fe - O distances and confirmed by bond valence calculation (Pyatenko, 1972).

### Crystal data

**Table:**

Cu_2.10_Fe_1.15_Li_4.59_(PO_4_)_4_	*Z* = 1
*M**_r_* = 609.63	*F*(000) = 293.9
Triclinic, *P*1	*D*_x_ = 3.480 Mg m^−^^3^
Hall symbol: -P 1	Mo *K*α radiation, λ = 0.71073 Å
*a* = 4.8950 (14) Å	Cell parameters from 8918 reflections
*b* = 7.847 (2) Å	θ = 2.4–28.3°
*c* = 8.388 (2) Å	µ = 5.87 mm^−^^1^
α = 69.472 (5)°	*T* = 298 K
β = 89.764 (6)°	Block, green
γ = 75.501 (5)°	0.27 × 0.23 × 0.19 mm
*V* = 290.88 (13) Å^3^	

### Data collection

**Table:**

Bruker SMART APEX diffractometer	1775 independent reflections
Radiation source: fine-focus sealed tube	1647 reflections with *I* > 2σ(*I*)
graphite	*R*_int_ = 0.023
φ scans, and ω scans	θ_max_ = 30.6°, θ_min_ = 2.6°
Absorption correction: multi-scan (*SADABS*; Bruker, 2000)	*h* = −7→7
*T*_min_ = 0.235, *T*_max_ = 0.332	*k* = −11→11
100441 measured reflections	*l* = −12→12

### Refinement

**Table:**

Refinement on *F*^2^	Primary atom site location: structure-invariant direct methods
Least-squares matrix: full	Secondary atom site location: difference Fourier map
*R*[*F*^2^ > 2σ(*F*^2^)] = 0.031	*w* = 1/[σ^2^(*F*_o_^2^) + (0.040*P*)^2^ + 0.5*P*] where *P* = (*F*_o_^2^ + 2*F*_c_^2^)/3
*wR*(*F*^2^) = 0.086	(Δ/σ)_max_ = 0.001
*S* = 1.20	Δρ_max_ = 0.66 e Å^−^^3^
1708 reflections	Δρ_min_ = −0.63 e Å^−^^3^
143 parameters	Extinction correction: *SHELXL97* (Sheldrick, 2010), Fc^*^=kFc[1+0.001xFc^2^λ^3^/sin(2θ)]^-1/4^
0 restraints	Extinction coefficient: 0.008 (3)

### Special details

**Table:**

Geometry. All e.s.d.'s (except the e.s.d. in the dihedral angle between two l.s. planes) are estimated using the full covariance matrix. The cell e.s.d.'s are taken into account individually in the estimation of e.s.d.'s in distances, angles and torsion angles; correlations between e.s.d.'s in cell parameters are only used when they are defined by crystal symmetry. An approximate (isotropic) treatment of cell e.s.d.'s is used for estimating e.s.d.'s involving l.s. planes.
Refinement. Refinement of *F*^2^ against ALL reflections. The weighted *R*-factor *wR* and goodness of fit *S* are based on *F*^2^, conventional *R*-factors *R* are based on *F*, with *F* set to zero for negative *F*^2^. The threshold expression of *F*^2^ > σ(*F*^2^) is used only for calculating *R*-factors(gt) *etc*. and is not relevant to the choice of reflections for refinement. *R*-factors based on *F*^2^ are statistically about twice as large as those based on *F*, and *R*- factors based on ALL data will be even larger.

### Fractional atomic coordinates and isotropic or equivalent isotropic displacement parameters (Å^2^)

**Table:**

	*x*	*y*	*z*	*U*_iso_*/*U*_eq_	Occ. (<1)
Cu1	−0.5000	1.0000	1.0000	0.00809 (14)	
Fe1	0.0000	1.0000	0.5000	0.00691 (15)	
Cu2	−0.09925 (13)	1.25406 (8)	0.71583 (7)	0.0080 (2)	0.551 (2)
Li2	−0.09925 (13)	1.25406 (8)	0.71583 (7)	0.0080 (2)	0.449 (2)
P1	−0.37011 (14)	1.70119 (10)	0.53149 (9)	0.00622 (16)	
P2	0.08696 (15)	0.80671 (10)	0.91211 (8)	0.00607 (16)	
Li1	0.3243 (15)	0.3973 (10)	1.1070 (9)	0.023 (2)	0.92 (3)
Fe2	0.3834 (13)	0.4186 (9)	0.8189 (9)	0.014 (2)	0.076 (3)
Li3	0.262 (2)	1.5161 (15)	0.7187 (13)	0.040 (2)	0.924 (3)
O1	0.2556 (4)	1.1578 (3)	0.4209 (3)	0.0106 (4)	
O2	0.3006 (4)	0.7643 (3)	0.5056 (3)	0.0090 (4)	
O3	0.2355 (5)	0.6208 (3)	0.8948 (3)	0.0132 (4)	
O4	−0.2135 (4)	0.7997 (3)	0.9644 (3)	0.0111 (4)	
O5	0.0858 (5)	0.9747 (3)	0.7461 (3)	0.0104 (4)	
O6	−0.2511 (4)	1.1666 (3)	0.9440 (2)	0.0076 (4)	
O7	0.2528 (4)	1.3249 (3)	0.6291 (3)	0.0108 (4)	
O8	−0.2947 (5)	1.5157 (3)	0.6891 (3)	0.0133 (4)	

### Atomic displacement parameters (Å^2^)

**Table:**

	*U*^11^	*U*^22^	*U*^33^	*U*^12^	*U*^13^	*U*^23^
Cu1	0.0056 (2)	0.0073 (2)	0.0128 (2)	−0.00214 (17)	0.00253 (17)	−0.00512 (18)
Fe1	0.0061 (3)	0.0056 (3)	0.0090 (3)	−0.00124 (19)	0.00114 (19)	−0.0029 (2)
Cu2	0.0102 (3)	0.0052 (3)	0.0078 (3)	−0.0007 (2)	0.0029 (2)	−0.0023 (2)
Li2	0.0102 (3)	0.0052 (3)	0.0078 (3)	−0.0007 (2)	0.0029 (2)	−0.0023 (2)
P1	0.0052 (3)	0.0056 (3)	0.0076 (3)	−0.0011 (2)	0.0007 (2)	−0.0023 (2)
P2	0.0054 (3)	0.0060 (3)	0.0067 (3)	−0.0009 (2)	0.0008 (2)	−0.0026 (2)
Li1	0.026 (4)	0.019 (4)	0.025 (4)	−0.002 (3)	−0.001 (3)	−0.011 (3)
Fe2	0.014 (3)	0.012 (3)	0.018 (4)	−0.004 (2)	0.006 (2)	−0.008 (3)
Li3	0.058 (6)	0.050 (6)	0.036 (5)	−0.024 (5)	0.013 (5)	−0.037 (5)
O1	0.0101 (9)	0.0133 (10)	0.0120 (9)	−0.0062 (8)	0.0023 (7)	−0.0069 (8)
O2	0.0052 (9)	0.0095 (9)	0.0134 (9)	−0.0009 (7)	−0.0001 (7)	−0.0063 (8)
O3	0.0153 (10)	0.0115 (10)	0.0134 (9)	0.0000 (8)	0.0017 (8)	−0.0079 (8)
O4	0.0066 (9)	0.0084 (9)	0.0186 (10)	−0.0015 (7)	0.0033 (8)	−0.0055 (8)
O5	0.0160 (10)	0.0091 (9)	0.0069 (8)	−0.0058 (8)	−0.0003 (7)	−0.0023 (7)
O6	0.0075 (9)	0.0101 (9)	0.0080 (8)	−0.0053 (7)	0.0013 (7)	−0.0044 (7)
O7	0.0105 (9)	0.0128 (10)	0.0127 (9)	−0.0055 (8)	0.0056 (7)	−0.0075 (8)
O8	0.0139 (10)	0.0088 (10)	0.0117 (9)	0.0013 (8)	0.0017 (8)	−0.0003 (8)

### Geometric parameters (Å, °)

**Table:**

Cu1—O4^i^	1.936 (2)	P2—O5	1.545 (2)
Cu1—O6	1.9430 (19)	P2—O6^v^	1.554 (2)
Fe1—O1	1.931 (2)	Li1—O3	1.967 (8)
Fe1—O5	2.038 (2)	Li1—O4^vi^	2.028 (7)
Fe1—O2	2.041 (2)	Li1—O8^v^	2.045 (7)
Cu2—O8	1.969 (2)	Li1—O3^vii^	2.124 (8)
Cu2—O7	1.998 (2)	Fe2—O3	1.891 (6)
Cu2—O6	2.003 (2)	Fe2—O8^viii^	2.063 (6)
Cu2—O5	2.075 (2)	Fe2—O7^ix^	2.133 (7)
Cu2—O2^ii^	2.171 (2)	Fe2—O6^viii^	2.236 (7)
P1—O7^iii^	1.521 (2)	Fe2—O4^vi^	2.334 (6)
P1—O1^iii^	1.524 (2)	Li3—O7	1.909 (8)
P1—O8	1.545 (2)	Li3—O3^x^	1.916 (7)
P1—O2^iv^	1.555 (2)	Li3—O2^x^	2.183 (11)
P2—O3	1.515 (2)	Li3—O8^xi^	2.183 (10)
P2—O4	1.542 (2)		
			
O4^i^—Cu1—O6	88.35 (9)	O5—Cu2—O2^ii^	78.90 (8)
O1—Fe1—O5^ii^	88.97 (8)	O7^iii^—P1—O1^iii^	111.94 (12)
O1—Fe1—O2	92.27 (9)	O7^iii^—P1—O8	112.64 (12)
O5^ii^—Fe1—O2	82.88 (8)	O1^iii^—P1—O8	106.40 (12)
O8—Cu2—O7	92.29 (9)	O7^iii^—P1—O2^iv^	109.52 (12)
O8—Cu2—O6	88.84 (9)	O1^iii^—P1—O2^iv^	110.70 (12)
O7—Cu2—O6	136.25 (9)	O8—P1—O2^iv^	105.43 (12)
O8—Cu2—O5	176.89 (9)	O3—P2—O4	108.60 (12)
O7—Cu2—O5	90.75 (9)	O3—P2—O5	111.10 (12)
O6—Cu2—O5	89.40 (9)	O4—P2—O5	112.78 (12)
O8—Cu2—O2^ii^	99.79 (9)	O3—P2—O6^v^	109.26 (12)
O7—Cu2—O2^ii^	102.81 (8)	O4—P2—O6^v^	108.23 (12)
O6—Cu2—O2^ii^	120.07 (8)	O5—P2—O6^v^	106.78 (11)

Symmetry codes: (i) −*x*−1, −*y*+2, −*z*+2; (ii) −*x*, −*y*+2, −*z*+1; (iii) −*x*, −*y*+3, −*z*+1; (iv) *x*−1, *y*+1, *z*; (v) −*x*, −*y*+2, −*z*+2; (vi) −*x*, −*y*+1, −*z*+2; (vii) −*x*+1, −*y*+1, −*z*+2; (viii) *x*+1, *y*−1, *z*; (ix) *x*, *y*−1, *z*; (x) *x*, *y*+1, *z*; (xi) *x*+1, *y*, *z*.
